# Decoupling Thermal and Hydraulic Performance in Cross-Flow Micro Heat Exchangers via Mixed-Geometry Channel Designs

**DOI:** 10.3390/mi17070776

**Published:** 2026-06-26

**Authors:** Quanyi Zhou, Zheng Chang, Qi Wang, Yuhao Dai, Lingjie Xu, Rongsheng Lin, Zenan Wu, Xianlei Chen, Wenfeng Wu

**Affiliations:** 1 School of Naval Architecture and Maritime, Zhejiang Ocean University, Zhoushan 316022, China; 2 Zhejiang Provincial Collaborative Innovation Center for Clean Energy Technology and Equipment in Marine Vessels, Zhoushan 316022, China; 3 Jurong Energy (Xinjiang) Co., Ltd., Urumqi 841603, China; 4 Zhoushan Institute of Calibration and Testing for Quality and Technology Supervision, Zhoushan 316022, China

**Keywords:** micro heat exchanger, thermal-hydraulic trade-off, mixed-geometry microchannels, microfluidics, corner flow dynamics, conjugate heat transfer

## Abstract

Cross-flow micro heat exchangers enable compact thermal management for high-density electronics, but their design is traditionally constrained by a strict trade-off between heat transfer and hydraulic resistance. To mitigate this limitation, we investigate the influence of mixed-geometry channel designs on the coupled thermal and hydraulic performance using a three-dimensional conjugate heat transfer model of water flowing through a stainless-steel micro-matrix with a 40-micrometer hydraulic diameter. Numerical simulations show that at low Reynolds numbers (100 to 200), corner-induced steady three-dimensional flow redistribution modifies the thermal boundary layer, causing convective and hydraulic performance to deviate from standard macroscale predictions. By expanding the transverse microchannel spacing from 10 to 60 μm, the Nusselt number increases from 1.15 to 2.07 while maintaining a nearly constant pressure gradient. These results provide geometric guidelines for designing high-efficiency microfluidic cooling systems by mitigating the traditional trade-off between heat-transfer enhancement and hydraulic resistance. Among the geometries evaluated, pure square channels maximize heat transfer, hybrid circular-square configurations optimize hydraulic efficiency, and triangular designs perform poorly due to high viscous drag. These results provide geometric guidelines for mitigating the traditional trade-off between heat-transfer enhancement and hydraulic resistance in microfluidic cooling systems.

## 1. Introduction

High-performance computing, artificial intelligence models, and heterogeneous computer architectures have significantly increased the integration density and thermal design power of electronic chips [1,2]. Advanced packaging techniques, such as 2.5D/3D stacking, generate high localized heat fluxes that exceed the limits of traditional air-cooling and macroscale heat exchangers, often triggering thermal throttling and reducing device reliability [3,4]. Microchannel heat sinks (MCHSs) provide a viable solution to these severe thermal constraints owing to their high surface-area-to-volume ratios and short thermal conduction paths [5,6]. Among microfluidic topologies, cross-flow micro heat exchangers (CFMCHEs) are especially suitable for space-constrained platforms, such as blade servers, because their orthogonal flow layouts simplify dense fluid routing while maintaining compact packaging [7,8,9]. Although macroscopic modifications such as double-layered channels [10] and manifold optimization [11] help mitigate global thermal gradients, the ultimate efficiency of these systems remains limited by microscale transport dynamics.

At the microscale, channel cross-sectional geometry dictates the coupling of velocity, pressure, and temperature fields [12]. Selecting these geometries requires balancing thermal-hydraulic performance against practical microfabrication limits. Building on comparative studies such as those by Ahmad et al. [13], this work evaluates four representative microchannel geometries: triangular, trapezoidal, hexagonal, and circular. The triangular channel serves to examine corner-induced flow redistribution and boundary-layer redevelopment; the trapezoidal and hexagonal channels represent intermediate polygonal profiles with distinct wetted perimeters; and the circular channel acts as a smooth, low-resistance reference. From a manufacturing perspective, triangular and trapezoidal microchannels can be fabricated via anisotropic wet etching of silicon, whereas micromachining and laser ablation enable the fabrication of hexagonal and circular profiles [14,15]. Assessing these geometries under identical hydraulic diameters clarifies how corner effects, wall curvature, and contact angles influence heat transfer and pressure drop—critical factors for data centers aiming to optimize thermal performance without excessive pumping power [16].

Square microchannels represent a common yet complex baseline for noncircular geometries [17]. Subject to uniform hydraulic diameter constraints, symmetric corners disrupt the thermal boundary layer and enhance transverse mixing at higher Reynolds numbers. However, under tight spatial constraints, these sharp corners introduce negative physical trade-offs. In viscosity-dominated laminar regimes, low-velocity fluid stagnates in the corners, leading to irregular wall shear stresses, high viscous dissipation, and elevated pumping power. Consequently, the thermal performance of square channels in cross-flow layouts depends heavily on whether localized mixing enhancements outweigh the associated pressure losses.

To mitigate pressure losses without adding complex internal features, researchers have investigated structural modifications like staggered baffles and interrupted channels [18,19]. More complex optimizations, including rhombic pin-fins and splitters, also improve multi-objective performance [20,21] by periodically disrupting the boundary layer. However, physically microfabricating internal solid barriers increases flow resistance and manufacturing complexity. Instead of adding internal microstructures, this study explores channel-profile reconfiguration as a passive, continuous flow-control strategy. By combining high-heat-transfer square sections with low-resistance, near-circular profiles in a mixed-geometry configuration, we seek to use geometry-induced flow redistribution to improve thermal transport while mitigating localized hydraulic resistance. Additionally, three-dimensional flow development in microchannels involves complex streamline redirection and orthogonal flow interference [22]. Entrance effects [23], surface confinement [24], and narrow geometry [25] can modify local streamline patterns and velocity distributions even at low Reynolds numbers. In orthogonally arranged square CFMCHEs [26], the physical relationships among corner-related velocity suppression, thermal-boundary-layer modification, non-linear Nusselt number variations, and deviations from classical laminar theory require further mechanism-level clarification based on viscous dissipation.

This study develops a three-dimensional conjugate heat transfer model to analyze steady laminar flow in square and mixed-geometry CFMCHEs. First, we quantify how square corners affect local velocity redistribution and thermal-boundary-layer development. Second, we assess the effects of channel count and transverse spacing on overall thermal-hydraulic performance. Finally, we compare several mixed-geometry configurations to demonstrate how transitioning to smoother cross-sections reduces pressure drop while preserving heat transfer effectiveness. This work offers design guidelines for resolving thermal-hydraulic trade-offs in compact microchannel liquid-cooling systems.

## 2. Numerical Methodology

### 2.1. Physical Model and Geometric Configurations

The physical object investigated in this study is a three-dimensional representative computational unit of a CFMCHE, which conceptualizes a highly compact localized cooling cell embedded within a high-heat-flux electronic chip thermal management substrate. While the full-scale heat sink comprises 20 periodically arranged units, a single representative core unit is extracted for the conjugate heat transfer (CHT) numerical simulations. As demonstrated in recent comparative structural studies on micro heat exchangers, this mathematical simplification dramatically reduces computational overhead while rigorously preserving the orthogonally coupled hot/cold fluid-solid heat transfer pathways, given that macroscopic edge effects are primarily confined to the outermost boundaries.

The bounding dimensions of the baseline representative computational domain are fixed at 800 μm in length, 120 μm in height, and 800 μm in width. Within this solid matrix, the embedded square microchannels feature a total length of 800 μm, with both the cross-sectional height and width fixed at 40 μm, yielding a constant equivalent hydraulic diameter of exactly 40 μm. To evaluate the influence of geometric parameters on thermal-hydraulic performance, the model is parameterized in terms of channel number and transverse channel spacing. The internal channel number *N_ch_* varies from 6 to 14, and the transverse channel spacing ranges from 10 μm to 60 μm. In addition, four mixed-channel configurations are constructed by combining the baseline square channels with triangular, trapezoidal, hexagonal, and circular profiles for comparative analysis. For all mixed-geometry configurations, the characteristic hydraulic diameter is kept constant at *D*_h_ = 40 μm. The cross-sectional dimensions of the triangular, trapezoidal, hexagonal, and circular channels are adjusted according to *D*_h_ = 4 Ac/Pw, where Ac is the channel cross-sectional area and Pw is the wetted perimeter. Therefore, the comparative evaluation isolates the influence of cross-sectional shape while avoiding artificial hydraulic-size effects.

Liquid water is employed as the single-phase coolant, while stainless steel is selected as the baseline solid substrate material. Although copper and aluminum possess higher thermal conductivities, excessive solid conductivity in compact microchannel heat exchangers can intensify longitudinal heat conduction, weaken the local temperature difference between the hot and cold streams, and reduce heat-exchanger effectiveness [27,28]. Stainless steel, with a thermal conductivity of approximately 15 W m^−1^ K^−1^, provides a moderate-conductivity matrix that limits axial heat leakage while maintaining conjugate heat-transfer capability. It also offers favorable mechanical robustness and corrosion resistance for liquid-cooled microstructures [29,30]. In this study, stainless steel is adopted as a fixed baseline material to isolate the effects of channel geometry. To replicate typical high-computing-power cooling scenarios, the inlet temperature of the hot stream is prescribed at 330 K, whereas the cold coolant stream is introduced at 300 K. The initial reference temperature of the entire computational domain is set at 293.15 K. At the fluid outlets, a static pressure boundary condition of 0 is applied. The uniform inlet velocities are directly derived from the predefined target *Re* to resolve both the low-velocity corner regions and the spatial evolution of steady three-dimensional flow structures. Additionally, the outer bounding surfaces of the computational unit are treated as adiabatic, assuming negligible thermal dissipation to the ambient environment.

### 2.2. Governing Equations and Assumptions

To establish a computationally efficient and physically consistent theoretical framework, several fundamental assumptions are adopted. Liquid water is modeled as a Newtonian continuum, while its thermophysical properties are not assumed to be constant. Instead, the density ρ(T), dynamic viscosity *μ*(T), specific heat capacity c_p_(T), and thermal conductivity *k*_f_(T) are evaluated as functions of the local temperature over the operating range of 300–330 K. This treatment explicitly accounts for the influence of local viscosity variation on the velocity profile, wall shear stress distribution, and thermal-boundary-layer development. Because the investigated Reynolds-number range remains low, the internal microfluidic flow is modeled as steady, laminar, and single phase. All flow features discussed in this work are interpreted within a steady laminar-flow framework. The Reynolds-number-dependent changes observed in the velocity and temperature fields are described as steady three-dimensional flow redistribution, secondary-flow development, and corner-induced flow restructuring, rather than as temporal transition, steady secondary-flow development, or turbulent instability. Gravitational effects, thermal buoyancy, and macroscopic body forces are neglected because surface and viscous forces dominate at these highly confined microscale dimensions. Internal viscous heating within the fluid domain and radiative thermal exchange along the solid boundaries are also neglected. The stainless-steel substrate is treated as a homogeneous and isotropic solid matrix.

The steady-state conjugate heat transfer within the computational domain is described by the three-dimensional, incompressible Navier–Stokes and energy equations for the laminar fluid phase, coupled with steady-state Fourier heat conduction in the solid substrate. At the fluid-solid interfaces, the coupling is closed by enforcing velocity no-slip, temperature continuity $\left(T_{f}\;=\;T_{s}\right)$, and heat-flux conservation $\;(-k_{f}\nabla T_{f}\cdot n\;=\;-k_{s}\nabla T_{s}\cdot n)$, where n represents the interface unit outward normal vector. These standard formulations are solved using temperature-dependent water properties, including density ρ(T), dynamic viscosity *μ*(T), specific heat capacity c_p_(T), and thermal conductivity *k*_f_(T), together with the isotropic thermal conductivity of the stainless-steel matrix, thereby ensuring strict global conservation of mass, momentum, and energy.

### 2.3. Mesh Generation and Grid-Independence Analysis

A rigorous grid independence study is performed using six sets of meshes with systematically varying densities to eliminate the discretization errors induced by the spatial mesh size. The comprehensive evaluation results, utilizing the average Nusselt number (*Nu*_avg_) and the total channel pressure drop under a baseline operating condition as the benchmark indicators, are systematically compiled in Table 1. Similar global or spatially averaged indicators, including the mean Nusselt number, total pressure drop, thermal resistance, and friction factor, have also been adopted in recent numerical studies of microchannels containing complex fins, ribs, porous structures, and sharp-edged flow disturbances [31,32]. The relative deviations between Mesh 5 and Mesh 6 are 0.10% for *Nu*_avg_ and 0.19% for the total pressure drop, confirming that further mesh refinement produces negligible changes in both thermal and hydraulic responses. Therefore, Mesh 5 is adopted for all subsequent simulations as a balanced choice between numerical accuracy and computational cost. For the mixed-geometry cases, the same refinement strategy was applied near the fluid–solid interfaces, corner regions, and hydrodynamic entrance zones to ensure consistent resolution of velocity, pressure, and temperature gradients. As the mesh density escalates from Mesh 1 to Mesh 6, both *Nu*_avg_ and the total pressure drop (Δp) gradually approach grid-independent values as the mesh density increases. When the mesh is refined from Mesh 4 to Mesh 5, the relative deviations for both the thermal performance indicator and the hydraulic resistance metric fall below 0.5%. Consequently, considering both the strict requirements for spatial resolution and computational economy, Mesh 5—comprising a total of 1,480,537 computational elements—is selected as the standard grid architecture for all subsequent parametric simulations.

The three-dimensional conjugate governing differential equations were numerically solved using the Galerkin finite element method in finite element analysis software. The CHT Multiphysics interface is invoked to intrinsically couple the laminar flow and solid heat conduction modules. To ensure numerical stability, suppress unphysical spatial oscillations in the convective transport terms, and accurately capture the steep gradients near the microchannel corners, consistent stabilization techniques including Streamline Upwind Petrov-Galerkin and Crosswind Diffusion are actively applied. Linear velocity and linear pressure or quadratic velocity and linear pressure elements are typically employed for the spatial discretization of the fluid domain to secure a high-order algorithmic fidelity. Double-precision computation is enabled across all cases. The iterative convergence of the numerical solution is determined by tracking the normalized residuals of the conservation equations. The convergence criteria are rigorously specified such that the scaled residuals for the continuity and momentum equations must fall below 10^−6^ while the residual for the energy equation must strictly terminate below 10^−8^, accompanied by the monitoring of a stabilized outlet temperature plateau.

### 2.4. Data Processing and Performance Evaluation Indicators

To rigorously evaluate and optimize the coupled thermal and hydraulic performance of the cross-flow micro heat exchangers across diverse geometric architectures, several critical dimensionless parameters and evaluation metrics are derived directly from the resolved computational fields.

The total heat-transfer rate ($\dot{Q}$) removed by the coolant stream is determined from the enthalpy balance of the fluid domain:

(1) $$\dot{Q}={\dot{m}}_{c}c_{p,c}(T_{c,\mathrm{out}}-T_{c,\mathrm{in}})={\dot{m}}_{h}c_{p,h}(T_{h,\mathrm{in}}-T_{h,\mathrm{out}})$$

where ${\dot{m}}_{c}$ and ${\dot{m}}_{h}$ represent the mass flow rates of the cold and hot streams, respectively; $T_{\mathrm{in}}$ and $T_{\mathrm{out}}$ denote the mass-weighted average temperatures at the respective inlet and outlet boundaries.

Where $\dot{Q}$ is the total heat-transfer rate, in W; ${\dot{m}}_{c}$ and ${\dot{m}}_{h}$ are the mass flow rates of the cold and hot streams, respectively, in *kgs*^−1^; $c_{p,c}$ and $c_{p,h}$ are the specific heat capacities of the cold and hot streams, respectively, in $Jkg^{-1}K^{-1}$; and $T_{c,\mathrm{in}}$, $T_{c,\mathrm{out}}$, $T_{h,\mathrm{in}}$, and $T_{h,\mathrm{out}}$ are the mass-weighted average temperatures at the corresponding inlet and outlet boundaries, in K.

The average convective heat transfer coefficient ($h_{\mathrm{avg}}$) at the fluid-solid interfaces is calculated as:

(2) $$h_{avg}=\frac{\dot{Q}}{A_{ht}\Delta T_{lm}}$$

where *h_avg_* is the average convective heat-transfer coefficient, in W m^−2^ K^−1^; $A_{\mathrm{ht}}$ is the total effective heat-transfer area of the microchannels, in m^2^; and $\Delta T_{lm}$ is the equivalent mean temperature difference, in K. Because the present CFMCHE adopts an orthogonal cross-flow arrangement, the uncorrected LMTD formulation is not used as the primary basis for evaluating global heat-exchanger performance. Instead, the heat-exchanger effectiveness is adopted as the primary global thermal-performance indicator and is calculated from the actual heat-transfer rate and the maximum possible heat-transfer rate, following the effectiveness-based framework commonly adopted for cross-flow heat exchangers [33]. For the calculation of the comparative average convective heat-transfer coefficient and average Nusselt number, an equivalent mean temperature difference is used only as a normalization parameter:

(3) $$\Delta T_{lm}=\frac{\left(T_{h,in}-T_{c,out})-(T_{h,out}-T_{c,in}\right)}{\ln\left(\frac{T_{h,in}-T_{c,out}}{T_{h,out}-T_{c,in}}\right)}$$

where $\Delta T_{lm}$ is the equivalent mean temperature difference, in K, and all inlet and outlet temperatures are expressed in K.

Consequently, *Nu_avg_*, which characterizes the non-dimensional convective heat-transfer intensity, is defined based on the constant channel hydraulic diameter ($D_{h}$) as follows:

(4) $$Nu_{avg}=\frac{h_{avg}D_{h}}{k_{f}}$$

where *Nu_avg_* is the average Nusselt number and is dimensionless; ($D_{h}$) is the hydraulic diameter of the microchannel, in m; and (*k_f_*) is the thermal conductivity of the fluid, in W m^−1^ K^−1^.

On the hydraulic side, the total pressure drop (Δp) across the channels is extracted directly from the static pressure difference between the inlet and outlet boundaries:

(5) $$\Delta p=p_{in}-p_{out}$$

where Δp is the total pressure drop, in Pa, and *P_in_* and *P_out_* are the mass-averaged static pressures at the inlet and outlet boundaries, respectively, in Pa.

To characterize the microscopic viscous shear resistance and evaluate deviations from classical fully developed laminar flow, the dimensionless friction factor (f) and Poiseuille number (Po) are introduced:

(6) $$\begin{array}{l} f=\frac{\Delta pD_{h}}{2\rho_{f}u_{avg}^{2}L}\\ Po=f\cdot Re \end{array}$$

where f is the friction factor and is dimensionless; Po is the Poiseuille number and is dimensionless; $\rho_{f}$ is the fluid density, in kg m^−3^; $u_{\mathrm{avg}}$ is the average fluid velocity inside the microchannels, in m s^−1^; *L* is the effective flow length of the channel, in m; and *Re* is the Reynolds number and is dimensionless.

Finally, to implement a multi-objective global optimization that concurrently accounts for heat transfer augmentation and hydraulic pumping penalties, the comprehensive Thermal-Hydraulic PEC is adopted:

(7) $$PEC=\frac{Nu_{avg}/Nu_{avg,0}}{{\left(Po/{Po}_{0}\right)}^{1/3}}$$

where PEC is the performance evaluation criterion and is dimensionless; $Nu_{\mathrm{avg},0}$ and ${Po}_{0}$ are the baseline average Nusselt number and baseline Poiseuille number of the pure square-channel configuration under the same operating condition, respectively, and are both dimensionless.

To evaluate the global hydrothermal efficiency of the mixed-geometry microchannels under a unified framework, several comprehensive metrics are further defined. The total pumping power required to drive both fluid streams is calculated as:

(8) $$P_{pump}={\dot{V}}_{h}\Delta P_{h}+{\dot{V}}_{c}\Delta P_{c}$$

where *P_pump_* is the total pumping power, in W; ${\dot{V}}_{h}$ and ${\dot{V}}_{c}$ are the volumetric flow rates of the hot and cold fluids, respectively, in m^3^ s^−1^; and $\Delta P_{h}$ and $\Delta P_{c}$ are the pressure drops of the hot-fluid and cold-fluid channels, respectively, in Pa.

Accordingly, the heat-exchanger effectiveness is used as the primary global thermal-performance indicator:

(9) $$\varepsilon =\frac{{\dot{Q}}_{actual}}{{\dot{Q}}_{max}}$$

where ε is the heat-exchanger effectiveness and is dimensionless; ${\dot{Q}}_{actual}$ is the actual heat-transfer rate between the hot and cold fluids, in W; and ${\dot{Q}}_{max}$ is the maximum theoretically possible heat-transfer rate, in W.

The pressure-drop-normalized thermal performance metric is calculated as:

(10) $$\eta =\frac{\varepsilon }{\Delta P}$$

where η is the pressure-drop-normalized thermal performance metric, in Pa^−1^; ε is the heat-exchanger effectiveness and is dimensionless; and ΔP is the total pressure drop across the microchannel system, in Pa. This metric evaluates the heat-exchanger effectiveness achieved per unit pressure-drop penalty.

The pumping-power-normalized hydraulic performance metric is calculated as:

(11) $$\eta^{*}=\frac{{\dot{Q}}_{actual}}{P_{pump}}$$

where $\eta^{*}$ is the pumping-power-normalized hydraulic performance metric and is dimensionless, expressed as W^−1^; ${\dot{Q}}_{actual}$ is the actual heat-transfer rate, in W; and *P_pump_* is the total pumping power required to drive the hot and cold fluids through the microchannels, in W. This metric represents the heat-transfer capacity achieved per unit pumping power.

### 2.5. Boundary Conditions and Solver Configurations

To obtain mathematically unique and physically consistent solutions for the conjugate governing equations, a well-posed set of boundary conditions is prescribed for all external and internal boundaries of the representative computational unit. At the hot- and cold-stream inlet boundaries, uniform velocity and constant-temperature conditions are specified. The hot-stream inlet temperature is maintained at *T*_h,in_ = 330 K, whereas the cold-stream inlet temperature is maintained at *T*_c,in_ = 300 K. The inlet velocity magnitude is adjusted according to the targeted Reynolds number and the constant hydraulic diameter. Meanwhile, the thermophysical properties of liquid water, including density, dynamic viscosity, specific heat capacity, and thermal conductivity, are evaluated as functions of the local temperature throughout the conjugate heat transfer domain.

At all fluid outlet boundaries, a pressure-outlet condition is imposed, with the operating static gauge pressure fixed at *P*_out_ = 0. At the internal fluid–solid conjugate interfaces where liquid water directly contacts the stainless-steel substrate, the hydrodynamic no-slip condition is enforced. The thermal coupling across the fluid–solid interface is governed by temperature continuity and heat-flux conservation, ensuring equal localized interface temperatures and uninterrupted conductive–convective heat transfer across the heterogeneous media. To isolate the core transport behavior and represent a periodic symmetric cell within the microchannel matrix, the top and bottom exterior bounding surfaces are defined as symmetry boundaries, while the remaining lateral outer surfaces of the solid matrix are prescribed as adiabatic walls to eliminate parasitic thermal exchange with the ambient environment.

Within the finite element analysis software, a stationary fully coupled solver is configured to solve the nonlinear fluid–thermal coupling system, with double-precision calculation enabled for all cases. The iterative solution is terminated according to strict convergence criteria. Specifically, the relative tolerance for all solved variables is required to fall below 10^−6^. In addition, global convergence is cross-verified by monitoring the mass-weighted average outlet temperature and the total channel pressure drop until both quantities reach stable, non-fluctuating plateaus. All reported thermal-hydraulic performance indicators, including the average Nusselt number, pressure drop, Poiseuille number, pumping power, heat-exchanger effectiveness, and performance evaluation criterion, are extracted from the converged solutions obtained using the temperature-dependent-property model.

## 3. Results and Discussion

### 3.1. Conjugate Heat Transfer Model Validation and Baseline Transport Characteristics

**Figure 1:**
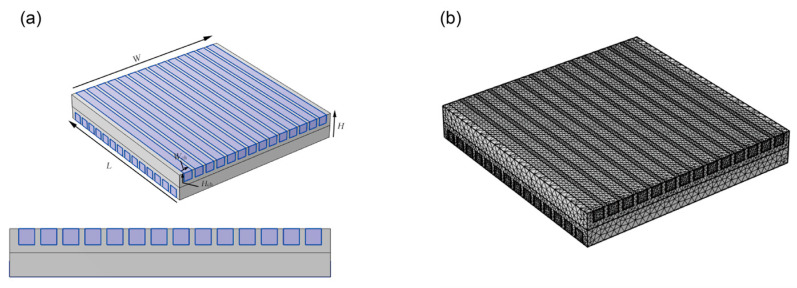
Representative computational domain and finite-element mesh discretization of the baseline square-channel CFMCHE: (**a**) three-dimensional orthogonal cross-flow layout highlighting the localized mesh refinement at the fluid-solid conjugate interfaces; (**b**) local cross-sectional view of the square microchannels embedded within the solid stainless-steel matrix.

Before conducting an architectural screening of optimized flow networks, a rigorous numerical validation and baseline characteristic analysis were performed using the pure square-channel CFMCHE matrix. As depicted in Figure 1a, the representative computational unit leverages a three-dimensional orthogonal layout where hot and cold microchannels are mutually perpendicular within a solid stainless-steel substrate. To eliminate spatial discretization errors and secure high-order algorithmic fidelity, a grid-independence study evaluating six distinct mesh densities, with the detailed finite-element mesh discretization illustrated in Figure 1b, was carried out under baseline operating conditions. As systematically quantified in Table 1, the average *Nu*_avg_ and global pressure drop Δp monotonically asymptotic toward a grid-independent plateau as the element count escalates. Upon transitioning from Mesh 4 to Mesh 5, the relative deviations for both the convective transport intensity and the hydraulic shear metric fall safely below 0.5%. Consequently, balancing spatial resolution requirements against computational economy, Mesh 5, comprising exactly 1,480,537 structured/unstructured elements, was selected as the standard grid architecture for all subsequent parametric simulations.

**Figure 2:**
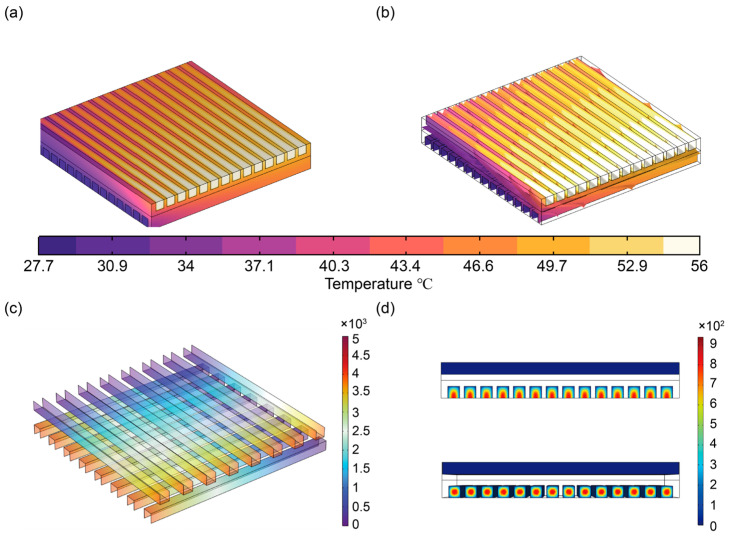
Baseline conjugate heat-transfer and hydrodynamic characteristics at *Re* = 20 (**a**,**b**) three-dimensional surface temperature distributions across the solid matrix and microchannel walls; (**c**) internal isothermal surfaces coupled with fluid streamline features; (**d**) local cross-sectional velocity and temperature fields illustrating high near-wall gradients and low-velocity regions near the channel corners.

The baseline transport phenomena governed by conjugate conduction through the solid carrier matrix and forced convection along the microchannel walls were evaluated at a representative low velocity (*Re* = 20), as visualized via the multi-physical field distributions in Figure 2. Specifically, the 3D solid surface temperature contours (Figure 2a,b) exhibit a highly continuous and smooth thermal gradient stretching from the hot passages (*T*_h,in_ = 330 K) toward the cold coolant streams (*T*_c,in_ = 300 K). The internal isothermal surfaces coupled with fluid streamline features (Figure 2c) are densely layered and concentrated around the spatial intersections of the orthogonal channels, establishing the dominance of multidirectional conjugate thermal pathways. The localized cross-sectional velocity vectors and temperature maps in Figure 2d identify low-velocity regions near the four corner vertices of the square microchannels. These stationary corner regions indicate that the sharp-cornered geometry modifies the local velocity and temperature fields under microscale confinement. The localized cross-sectional velocity vectors and temperature maps in Figure 2d identify low-velocity regions near the four corner vertices of the square microchannels. These low-velocity regions are interpreted only as evidence of local velocity suppression and velocity-field non-uniformity under microscale confinement, rather than as direct evidence of secondary flow or recirculation. The hydraulic and thermal influence of the corner regions is further evaluated in Section 3.2 using the Poiseuille number and Nusselt number.

### 3.2. Quantitative Analysis of Corner-Induced Viscous Resistance and Boundary-Layer Disturbance

**Figure 3:**
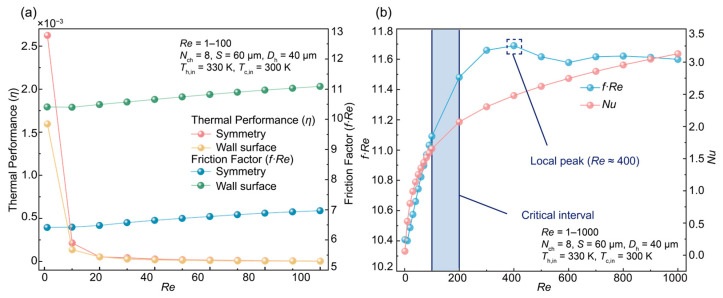
Quantitative verification of corner effect and spatial evolution of steady flow structure: (**a**) comparison of thermal performance and Po under symmetry and physical wall boundaries; (**b**) evolution of *Nu* and *Po* with *Re* up to 1000, showing spatial flow-structure evolution interval *Re* between 100 and 200.

To avoid repeated qualitative interpretation of the corner effect, the dual role of sharp square vertices is quantitatively analyzed in this subsection. The hydrodynamic contribution of the corners was isolated by comparing the fully enclosed physical-wall configuration with an idealized symmetry-boundary case. As shown in Figure 3a, removing the wall-bounding corner constraints reduces the Poiseuille number to approximately 6.4–7.0. This reduction directly quantifies the viscous shear penalty introduced by the sharp-cornered geometry under steady laminar microscale flow conditions. The thermal consequence of the same corner-induced flow structure is evaluated using the coupled variation of the average Nusselt number and Poiseuille number in Figure 3b. When the Reynolds number increases to the interval of *Re* between 100 and 200, the flow field exhibits steady three-dimensional velocity redistribution and corner-induced flow restructuring within the square microchannel. These structures disturb the thermal boundary layer and promote convective heat transfer, while also increasing viscous resistance. The local peak of (*Po*), approximately 11.7 near *Re* = 400, further indicates that the hydraulic penalty increases rapidly once the corner-induced flow redistribution becomes stronger.

Therefore, the sharp-cornered geometry should be understood as producing a coupled but competing thermal-hydraulic effect. On the one hand, corner-induced flow restructuring modifies the local thermal boundary layer and enhances local convective transport. On the other hand, the same corner regions intensify wall shear and increase the Poiseuille number, thereby increasing pressure drop and pumping-power demand. This quantitative evidence provides the reference mechanism for interpreting the geometric optimization results discussed in Section 3.3 and Section 3.4.

### 3.3. Multi-Geometry Mixed-Channel Design and Localized Flow-Field Response

**Figure 4:**
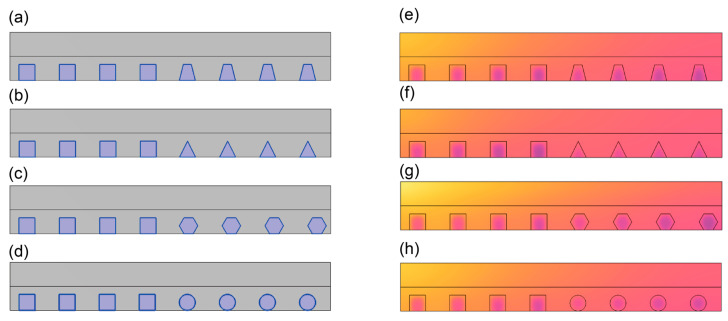
Two-dimensional cross-sectional geometric layouts and corresponding localized thermal fields of the multi-geometry mixed-channel CFMCHEs. Panels (**a**–**d**) illustrate the structural arrangements integrating the baseline square microchannels (left side) with different hybrid profiles (right side): (**a**) trapezoidal, (**b**) triangular, (**c**) hexagonal, and (**d**) circular channels. Panels (**e**–**h**) display the respective steady-state cross-sectional temperature distributions corresponding to the geometries above: (**e**) square-trapezoidal, (**f**) square-triangular, (**g**) square-hexagonal, and (**h**) square-circular hybrid configurations. The thermal maps visually demonstrate how smooth boundaries promote continuous and uniform thermal penetration, whereas sharp-cornered configurations induce localized thermal gradients.

To break the rigid hydraulic performance bottleneck of pure square channel networks without introducing complex solid baffling structures, a smooth geometric reconfiguration strategy was implemented. This approach utilizes the square cross-section as the baseline high-heat-transfer unit while introducing smoother, multi-geometry mixed channels (triangular, trapezoidal, hexagonal, and circular profiles) as synergistic partners to relieve localized viscous resistance. Figure 4 illustrates the two-dimensional cross-sectional layouts (Figure 4a–d) and the resulting localized thermal fields of these heterogeneous mixed-geometry configurations. Specifically, the structural arrangements pair the baseline square channels with trapezoidal (Figure 4a), triangular (Figure 4b), hexagonal (Figure 4c), and circular (Figure 4d) profiles. The thermal maps reveal that smooth or near-smooth boundaries, as prominently seen in the hexagonal (Figure 4g) and circular (Figure 4h) hybrids, distribute the temperature fields more continuously and uniformly along the channel perimeter. As shown in Figure 4e,f, the circular-hybrid configuration presents a more continuous and uniform temperature distribution, whereas the triangular-hybrid configuration exhibits more localized and non-uniform thermal fields, indicating less favorable heat spreading within the surrounding substrate. In contrast, the triangular-hybrid configuration exhibits more non-uniform local thermal fields, indicating less favorable heat spreading within the surrounding substrate.

**Figure 5:**
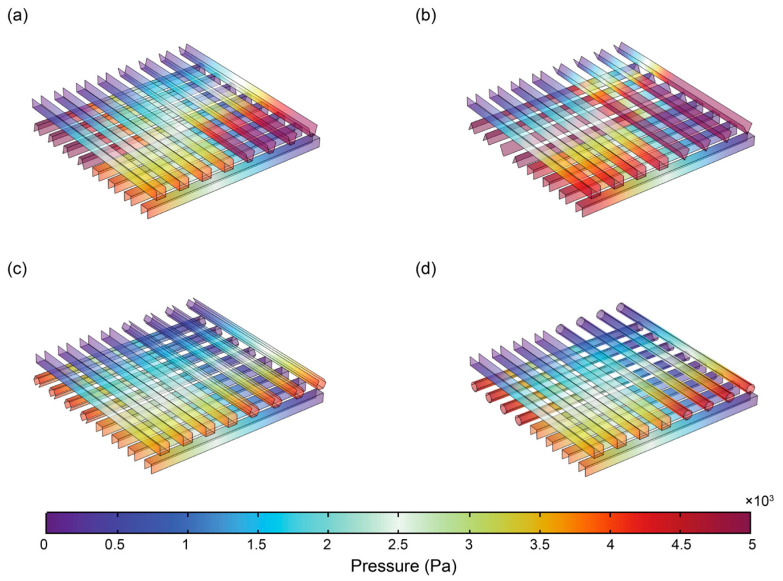
Three-dimensional static pressure field distributions of the heterogeneous mixed-channel CFMCHEs under optimized geometric configurations. The panels explicitly compare the fluidic pressure contours across four distinct hybrid profiles coupled with the baseline square channels: (**a**) square-trapezoidal, (**b**) square-triangular, (**c**) square-hexagonal, and (**d**) square-circular configurations. The color maps visually underscore the severe localized pressure concentrations and extreme hydraulic penalties induced by flow separation at the acute vertices of the triangular profile in (**b**). In stark contrast, the smooth, streamlined boundaries of the near-circular and circular channels in (**c**,**d**) effectively relieve viscous dissipation and minimize global flow resistance.

**Figure 6:**
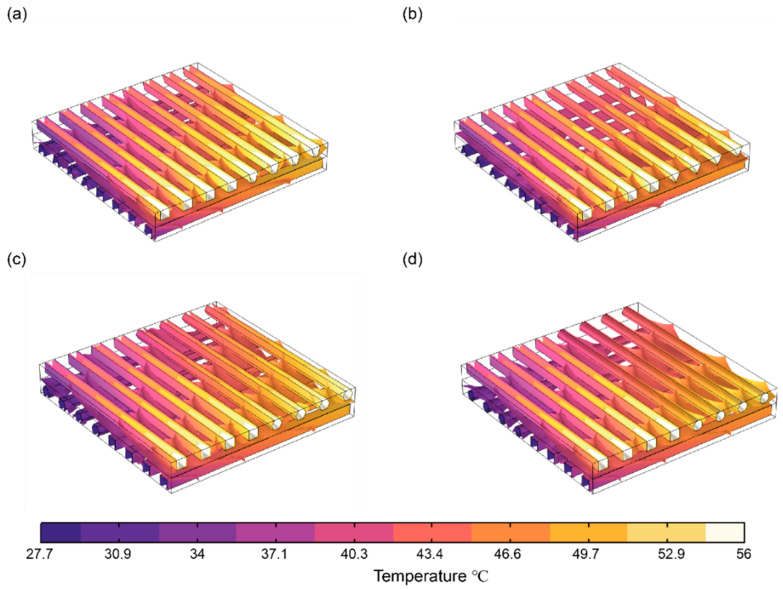
Three-dimensional conjugate temperature field distributions within the heterogeneous mixed-channel CFMCHEs under optimized geometric configurations. The panels illustrate the spatial thermal uniformity across four distinct hybrid profiles coupled with the baseline square channels: (**a**) square-trapezoidal, (**b**) square-triangular, (**c**) square-hexagonal, and (**d**) square-circular configurations. The thermal contours visually demonstrate that configurations with smooth boundaries or well-matched structural profiles, particularly the circular hybrid in (**d**), facilitate highly uniform wall-contact areas and continuous, symmetric heat flux distributions. Conversely, the mismatched and excessively sharp vertices of the triangular profile in (**b**) inherently degrade the spatial uniformity of conjugate heat conduction, resulting in constrained thermal penetration within the solid carrier matrix.

The underlying physical mechanisms of these configuration-dependent transport properties are thoroughly explained by the three-dimensional pressure and temperature field reconstructions shown in Figure 5 and Figure 6. As emphasized by the 3D pressure contours (Figure 5), the triangular hybrid channel (Figure 5b) produces the strongest localized pressure concentrations and severe global friction accumulation compared to the trapezoidal configuration (Figure 5a). This is because its sharp, acute-angled vertices force extensive flow separation, inducing intense viscous dissipation and low-velocity flow stagnation within the corners. In sharp contrast, the hexagonal (Figure 5c) and circular (Figure 5d) hybrid configurations smoothly guide the fluid streamlines along the near-wall curvature, successfully suppressing flow separation and significantly relieving the localized pumping pressure penalties. Thermally, as visualized in Figure 6, the circular (Figure 6d) and square-based passages maintain highly uniform wall-contact areas and continuous heat flux distributions. The hexagonal configuration (Figure 6c) also exhibits well-distributed thermal fields, closely approaching the performance of the circular channels. This structural matching ensures symmetric, uninterrupted energy balance pathways throughout the heterogeneous computational domain, whereas mismatched or excessively sharp geometries, most notably the triangular profile (Figure 6b), inherently degrade the spatial uniformity of conjugate heat conduction within the solid carrier matrix.

### 3.4. Comprehensive Thermal-Hydraulic Evaluation and Global Optimization

**Figure 7:**
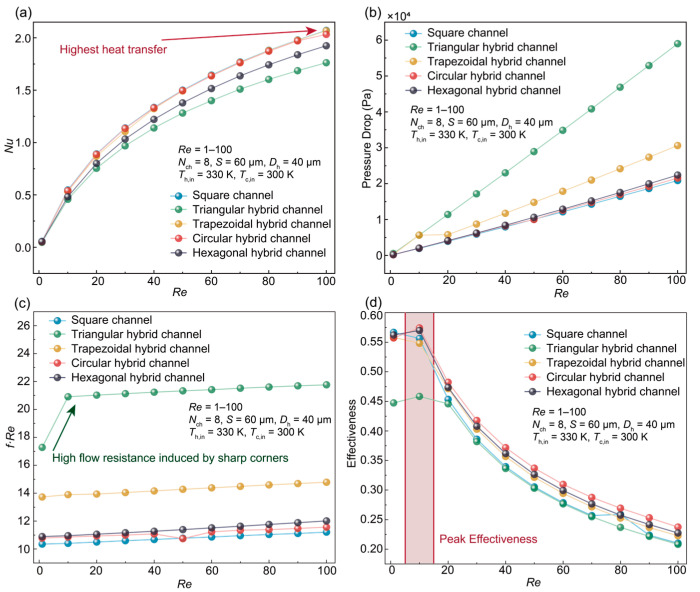
Comprehensive thermal-hydraulic performance comparison of the baseline square and mixed-channel CFMCHEs as a function of Reynolds number (*Re* = 1–100): (**a**) average Nusselt number (*Nu*); (**b**) global pressure drop (Δ*p*); (**c**) Poiseuille number (*Po* = *f*·*Re*); (**d**) heat-exchanger effectiveness (ε).

To systematically evaluate the overall performance trends, a performance screening was conducted over a wide operating range (*Re* = 1–100) under the optimized geometric constraints (*N_ch_* = 8, channel spacing = 60 μm). In the present analysis, *Nu* is used to compare the average convective heat-transfer intensity, whereas ε is used to evaluate the global heat-exchanger effectiveness of the cross-flow configuration. As plotted in Figure 7a, *Nu* increases monotonically with Re for all geometries, which is consistent with forced-convection enhancement. The square, circular-hybrid, and trapezoidal-hybrid configurations show higher heat-transfer levels, whereas the triangular-hybrid configuration exhibits the lowest overall heat-transfer performance. On the hydraulic side, Figure 7b,c show that the triangular-hybrid channel produces the largest pressure drop and Poiseuille number, while the circular-hybrid channel maintains the lowest hydraulic resistance over the tested Reynolds-number range. Meanwhile, the heat-exchanger effectiveness *ε* decreases with increasing *Re* for all geometries because of the reduced fluid residence time at higher velocities; nevertheless, the circular-hybrid configuration maintains higher effectiveness over most of the tested range.

**Figure 8:**
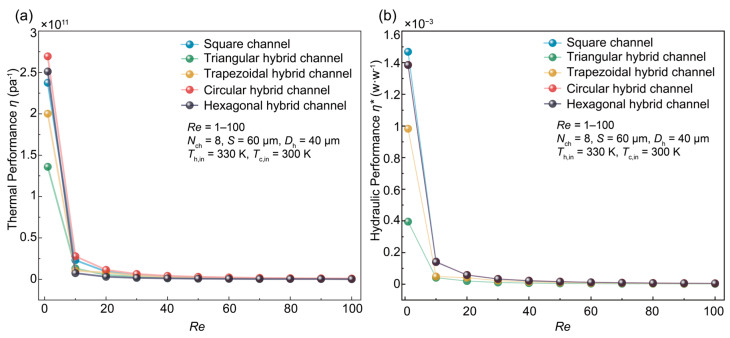
Multi-objective global optimization based on integrated performance indicators for the evaluated CFMCHE configurations: (**a**) comprehensive thermal performance index (*η*); (**b**) comprehensive hydraulic performance index (η*), identifying the optimal trade-off between heat transfer augmentation and parasitic pumping power.

To achieve a multi-objective global optimization that balances thermal augmentation against parasitic pumping power penalties, the comprehensive thermal performance (*η*) and hydraulic performance (η*) indicators are integrated and compared in Figure 8a and Figure 8b, respectively. As illustrated in Figure 8a, the pure square-channel matrix provides the highest comprehensive thermal performance index *η* among the evaluated configurations, mainly due to its larger effective wall-contact area. In contrast, Figure 8b shows that the circular-hybrid channel achieves the highest comprehensive hydraulic performance index η*, reflecting its lower pressure-drop penalty and reduced pumping-power requirement. The triangular-hybrid channel performs poorly in both comprehensive thermal and hydraulic indicators, indicating that its hydraulic penalty is not compensated by a corresponding thermal benefit within the tested Reynolds-number range.

Parametric optimization of the internal channel number *N*_ch_ from 6 to 14 shows that increasing the number of channels enlarges the effective convective surface area and improves the heat-exchanger effectiveness under the prescribed geometric constraints. Systematically varying the transverse channel spacing from 10 μm to 60 μm reveals a partial separation between the dominant thermal and hydraulic responses within the investigated design range. As the inter-channel spacing increases, the lateral conductive thermal resistance within the solid carrier matrix decreases substantially, enabling heat to be conducted more efficiently toward the fluid-solid interface. Consequently, the thermal effectiveness of the square microchannel configuration increases by 46.15%, a gain mainly associated with reduced solid-phase conduction resistance and improved heat spreading within the solid matrix. This improvement in thermal performance occurs without a comparable increase in global pressure drop, indicating that transverse spacing mainly affects solid-phase heat-spreading pathways rather than the hydraulic flow resistance under the present geometric constraints. These results indicate that transverse spacing provides a geometric parameter for improving thermal performance with limited influence on hydraulic resistance under the present operating conditions. The thermal improvement is mainly associated with modified solid-phase conduction pathways and enlarged effective heat-spreading regions, whereas the hydraulic resistance remains nearly unchanged because the channel hydraulic diameter and flow path are preserved. Therefore, the observed behavior should be interpreted as trade-off mitigation within the present design space, rather than as independent control of heat transfer and pressure drop.

Collectively these insights establish a robust geometric design framework for advanced microfluidic thermal management. When absolute heat extraction remains the primary design constraint deploying a pure square channel architecture provides the highest thermal dissipation capacity. Conversely integrating spacing tuned heterogeneous circular geometries yields a highly balanced and energy-efficient fluidic network. This optimized architectural pathway effectively resolves persistent hydraulic bottlenecks, thereby meeting the stringent energy efficiency demands inherent to next generation high performance computing facilities. Collectively, these results provide geometry-based design guidance for cross-flow micro heat exchangers. When heat-transfer enhancement is the primary objective, the square-channel architecture provides the highest thermal performance. When hydraulic efficiency and pumping-power reduction are prioritized, the circular-hybrid configuration offers a more favorable compromise. Therefore, mixed-geometry channel design provides a practical route to mitigate, rather than completely eliminate, the traditional trade-off between heat-transfer enhancement and hydraulic resistance.

## 4. Conclusions

This study establishes a rigorous three-dimensional conjugate heat transfer model based on the Galerkin finite element method to systematically evaluate and optimize micro heat exchangers for high-density electronics cooling. By linking microscopic steady flow-structure evolution with macroscopic geometric architectures, we unveil the fundamental flow mechanisms and structural design principles governing these advanced microfluidic devices.

Analyzing the fundamental transport characteristics reveals that the sharp internal vertices of baseline square microchannels dictate a complex dual physical effect. The analysis shows that sharp internal vertices affect both viscous resistance and convective transport, as quantified by the Poiseuille and Nusselt numbers in Section 3.2. This interaction produces Reynolds-number-dependent steady three-dimensional flow redistribution within the low-velocity interval of *Re* between 100 and 200. This steady flow redistribution is characterized by nonlinear variations in the Nusselt number and a corresponding peak in the Poiseuille number near *Re* = 400.

Beyond these fluid dynamics we discovered a profound thermal and hydraulic decoupling mechanism driven by spatial geometric parameters. Parametric evaluations across channel counts ranging from six to fourteen demonstrate that expanding the convective surface area monotonically augments thermal effectiveness. The pure square configuration exhibits superior area sensitivity, achieving a relative growth rate exceeding 21 percent alongside a highly competitive internal pressure drop. More importantly expanding the transverse channel spacing from 10 micrometers to 60 micrometers drastically reduces the lateral conductive thermal resistance within the solid matrix. This structural modification elevates the thermal effectiveness of the square architecture by over 46 percent while maintaining a strictly invariant global pressure gradient.

Building upon these spatial decoupling principles a universal geometric screening across six distinct structural configurations clarifies the crucial role of cross-sectional shapes. Designs featuring sharp corners suffer from severe localized stress concentrations and excessive viscous dissipation penalties. Conversely incorporating smoother near-circular boundaries significantly streamlines localized velocity profiles and relieves viscous drag. Integrating these low-resistance boundary contours into a highly convective solid matrix helps mitigate primary hydraulic bottlenecks. This geometric strategy provides an energy-efficient architectural solution that provides a practical route to mitigate, rather than completely eliminate, the traditional trade-off between heat-transfer enhancement and pumping-power consumption.

Ultimately transitioning from conventional uniform square matrices to spacing tuned heterogeneous mixed geometry flow networks provides geometry-based guidance for microfluidic device design. These geometric insights provide a rigorous and scalable foundation for advancing the thermal management of future green data centers and high-performance computing infrastructures.

## Figures and Tables

**Table 1:** Grid independence verification metrics at *Re* = 100.

Mesh Index	Total Element Number	Average Nusselt Number	Total Pressure Drop	Relative Deviation	Relative Deviation
Mesh 1	420,351	1.821	142.3	—	—
Mesh 2	685,412	1.954	151.8	7.30%	6.68%
Mesh 3	954,128	2.012	156.4	2.97%	3.03%
Mesh 4	1,235,471	2.051	159.2	1.94%	1.79%
Mesh 5	1,480,537	2.058	159.8	0.34%	0.38%
Mesh 6	1,854,210	2.060	160.1	0.10%	0.19%

## Data Availability

The original contributions presented in this study are included in the article. Further inquiries can be directed to the corresponding author.
